# Deletion of the *gabra2* Gene Results in Hypersensitivity to the Acute Effects of Ethanol but Does Not Alter Ethanol Self Administration

**DOI:** 10.1371/journal.pone.0047135

**Published:** 2012-10-24

**Authors:** Claire I. Dixon, Sophie E. Walker, Sarah L. King, David N. Stephens

**Affiliations:** School of Psychology, University of Sussex, Brighton, United Kingdom; McLean Hospital/Harvard Medical School, United States of America

## Abstract

Human genetic studies have suggested that polymorphisms of the GABRA2 gene encoding the GABA_A_ α2-subunit are associated with ethanol dependence. Variations in this gene also convey sensitivity to the subjective effects of ethanol, indicating a role in mediating ethanol-related behaviours. We therefore investigated the consequences of deleting the α2-subunit on the ataxic and rewarding properties of ethanol in mice. Ataxic and sedative effects of ethanol were explored in GABA_A_ α2-subunit wildtype (WT) and knockout (KO) mice using a Rotarod apparatus, wire hang and the duration of loss of righting reflex. Following training, KO mice showed shorter latencies to fall than WT littermates under ethanol (2 g/kg i.p.) in both Rotarod and wire hang tests. After administration of ethanol (3.5 g/kg i.p.), KO mice took longer to regain the righting reflex than WT mice. To ensure the acute effects are not due to the *gabra2* deletion affecting pharmacokinetics, blood ethanol concentrations were measured at 20 minute intervals after acute administration (2 g/kg i.p.), and did not differ between genotypes. To investigate ethanol’s rewarding properties, WT and KO mice were trained to lever press to receive increasing concentrations of ethanol on an FR4 schedule of reinforcement. Both WT and KO mice self-administered ethanol at similar rates, with no differences in the numbers of reinforcers earned. These data indicate a protective role for α2-subunits, against the acute sedative and ataxic effects of ethanol. However, no change was observed in ethanol self administration, suggesting the rewarding effects of ethanol remain unchanged.

## Introduction

Human genetic studies have suggested that polymorphisms of the GABRA2 gene encoding the GABA_A_ α2-subunit are associated with ethanol dependence in European American, white American, American plains Indian tribe and Russian populations [1], [2], [3], [4], though, unsurprisingly, this association is not evident in all populations [5], [6]. Although γ-aminobutyric acid A (GABA_A_) receptors have been suggested to represent a primary target for ethanol, the direct effects of ethanol at postsynaptic receptors are achieved only at high concentrations unlikely to be achieved by social drinkers (see [7] for review). Lower concentrations of ethanol can, however, can affect inhibitory GABAergic transmission by increasing release of GABA [8] and the release of neuromodulators which are active at the GABA_A_ receptor, most notably neurosteroids [9].

Since Edenberg *et al*’s original report [2], related or identical variants in GABRA2 genes have also been associated with other addictive behaviours, including cocaine abuse [10], heroin abuse [11] and polydrug abuse [12], [13]. That related haplotypes were associated with several forms of addiction make it unlikely that possession of a risk haplotype simply confers increased or decreased sensitivity to ethanol. Indeed the same variations are also associated with childhood conduct disorder [14], [15] and with increased impulsivity [16], behavioural traits that may contribute to the development of addictive behaviours. There is also emerging evidence that the influence of GABRA2 haplotypes on the development of addictions is due to an interaction with early life stress [11]. Since facilitated GABAergic transmission via α2-subunit containing receptors contributes to the anxiolytic action of benzodiazepines [17], [18] and barbiturates [19] and deletion of the α2-subunit gives rise to an anxious phenotype [19], genetic variations may contribute to the ability to cope with early life stress, and thus lead to an increased likelihood of addiction.

Nevertheless, there is also evidence that the same haplotypic variations in GABRA2 may alter the subjective effects of ethanol ingestion as measured by self-assessment of ethanol-related sensations [20] and mood [21], and studies using α2-subunit mutant mice suggest that alcohol consumption is influenced by the manipulation even at socially relevant concentrations of ethanol [22]. These findings suggest a more direct relationship between ethanol and α2-subunit containing GABA_A_ receptors and may be resolved by considering the role of intermediate neurotransmitters. For example, inhibition of neurosteroid synthesis (which is activated by ethanol) attenuates differences between risk and protective haplotypes [23], suggesting that ethanol may indirectly facilitate transmission at α2-subunit containing receptors by increasing levels of neurosteroids.

Animal experiments using manipulations of the α2-subunit *in vivo* have also suggested a more direct GABA-ethanol interaction but currently yield somewhat contradictory results. Using a knockout mouse model, deletion of the α2-subunit resulted in decreased sedation in response to an acute ethanol challenge, as measured by the loss of righting reflex [24], suggesting that the α2-subunit conveys sensitivity to the sedative effects of ethanol (though, curiously, it mediates stimulant effects of benzodiazepines [25], [26]). Mutations of the α2-subunit (S270I in transmembrane region 2 (TM2), A291W in TM3 [27] and α2(S270H, L277A) [22]) result in decreased ethanol potentiation of the GABA response in *Xenopus* oocytes. Mice bearing the latter mutation show reduced ethanol-induced locomotor activation and a corresponding increase in sedative effects as measured by loss of righting reflex [22]. Such results appear inconsistent with a decreased ethanol potentiation of GABA responses and with the behavioural data reported in knockout mice. Nevertheless these apparently contradictory data do suggest a potential role for the GABA_A_ α2-subunit in several mechanisms that may contribute to the development of dependence to ethanol, including acute subjective responses to ethanol.

We therefore investigated the role of the GABA_A_ α2-subunit in ethanol related behaviours using a knockout mouse model. Acute effects were investigated using tests of sedation, locomotor activation and motor coordination, whilst motivation to consume ethanol was tested using an operant oral self administration procedure.

## Methods

### Ethics Statement

All experiments were carried out with a UK Home Office project licence under the authority of the UK Animals (Scientific Procedures) Act 1986 and in accordance with local guidelines. Experiments were performed at the University of Sussex, which is a designated facility according to the Act. Following the experiments, except where stated otherwise, mice were killed by CO2 inhalation which is an appropriate method under Schedule 1 of the UK Animals (Scientific Procedures) Act 1986.

### Animals

Wildtype (WT) and knockout (KO) male mice were bred from heterozygous pairings and maintained on a mixed 50% C57BL/6J –50% 129SvEv background. α2-subunit KO mice were generated as previously described [19]. Animals were housed in pairs under a 12 hr light/dark cycle (lights on at 7.00 AM) in a holding room with controlled temperature (≈21°C) and humidity (≈50%). Except where specified, animals had *ad libitum* access to standard laboratory chow (Bekay Feeds, Hull, UK) and water within the home cage. Mean body weight at the start of the experiment was approximately 30 g.

### Drugs

Ethanol (95%) was diluted in 0.9% saline to a concentration of 20% v/v and administered i.p. at varying volumes (maximum 20 ml/kg) to achieve the required dose.

### Sedative and Ataxic Effects of Acute Administration

#### Rotarod

To assess motor coordination, WT and KO mice (n = 10) were trained to remain on a rotating rod apparatus (Rotarod; Ugo Basile, Comerio, Italy), revolving at 16 revolutions per minute. Each animal had two 5 minute training sessions. The following day, all animals were re-tested for their ability to complete a 3 minute trial prior to drug administration. Animals were injected with 2 g/kg ethanol and tested on the Rotarod at 20 minute intervals until two consecutive 3 minute trials were completed without falling.

*Statistical analysis.* A two-way, repeated measures ANOVA, with the between subject factor genotype and within subject factor timepoint was used to evaluate potential genotype and ethanol effects for the latency to fall, using SPSS statistics package. The Greenhouse-Geisser correction was applied to correct for a violation in the assumption of sphericity.

#### Wire hang

To assess motor strength, WT and KO mice (n = 12) were trained to grip and hang onto a taut wire suspended 50 cm above the bench top. Training was considered complete when each animal maintained grip on the wire for 60 seconds on two occasions. The following day, all animals were injected with 2 g/kg ethanol and their ability to hang from the wire, as measured by latency to fall, was recorded prior to and at 20, 40 and 60 minutes post-injection.

#### Statistical analysis

A two-way, repeated measures ANOVA with the between subject factor genotype and within subject factor timepoint was used to evaluate potential genotype and ethanol effects for the variable latency to fall. Data were log transformed to correct for a violation of homogeneity of variance.

#### Loss of righting reflex

To assess the sedative effects of ethanol, WT and KO mice (n = 12) were injected with 3.5 g/kg ethanol. At intervals of one minute, mice were tested for their ability to recover an upright posture within 10 seconds. The time of loss of righting reflex and duration of loss were recorded.

#### Statistical analysis

Duration of loss of righting reflex was compared between the genotypes using an independent samples Student’s t-test.

#### Locomotor activity

After 20 minute habituation sessions to the locomotor runways for 2 days, WT and KO mice (n = 8) were injected i.p. with 0, 1, 2 or 3 g/kg and immediately placed in circular runways to record locomotor activity for 5 minutes. Ethanol doses were administered in a counterbalanced design, at 2 day intervals.

#### Statistical Analysis

Habituation data were analysed using a two-way, repeated measures ANOVA comparing genotype and habitation day. To determine ethanol effects on locomotor activity, a two-way, repeated measures ANOVA with the between subject factor genotype and within subject factor dose was used to evaluate potential genotype and ethanol effects on locomotor activity.

### Motivation to Obtain Ethanol

#### Operant self administration

WT and KO mice (n = 8) were food restricted to reduce body weights to 90% of free-feeding weight, and trained to press a lever for 10% sucrose in mouse operant chambers (Med Associates, Georgia, VT, USA) constructed of clear Perspex (18×18×15 cm), and contained in sound and light attenuating cubicles. Each operant chamber possessed a single house light located on the wall opposite the levers. The front wall was fitted with a liquid dipper, located between 2 ultrasensitive mouse levers. Following an initial 15 hour training session, which included the dark phase, the response requirement to obtain 10% sucrose reinforcer was increased over consecutive days (range 2–7) from FR1 to FR2 to FR4 in daily one hour sessions. Motivation to consume ethanol was investigated using the sucrose fading technique [28]: over the following sessions the sucrose concentration was decreased and the ethanol concentration was increased as follows; 10% sucrose +3% ethanol; 10% sucrose +5% ethanol, 7% sucrose +5% ethanol, 10% sucrose +10% ethanol. The mice remained at each stage until reaching a criterion of at least 30 reinforcers per session on 3 consecutive days.

#### Statistical analysis

Sessions to criteria at each sucrose/ethanol concentration were analysed using a Mann-Whitney U test. A two-way, repeated measures ANOVA, with the between subject factor genotype and within subject factor ethanol/sucrose concentration was used to evaluate potential genotype and reinforcer effects for the number of lever presses and number of reinforcers obtained. The Greenhouse-Geisser correction was applied to correct for a violation in the assumption of sphericity.

### Blood Ethanol Concentration

WT and KO mice (n = 4 per time point) were administered ethanol (2 g/kg, i.p.) acutely. At 15, 45 and 120 minutes following administration, mice were sacrificed by cervical dislocation and trunk blood collected into heparinised capillary tubes. Blood samples were centrifuged for 10 minutes and supernatant frozen at −80°C. Samples were analysed in triplicate using an Analox automatic ethanol analyser (AM1, Analox Instruments, London, UK) and the mean value taken for statistical analysis.

#### Statistical analysis

A two-way, repeated measures ANOVA, with the between subject factors genotype and sampling time was used to evaluate potential genotype and timepoint effects for the variable ethanol level.

## Results

### Sedative and Ataxic Effects of Acute Administration

#### Rotarod

**Figure 1:**
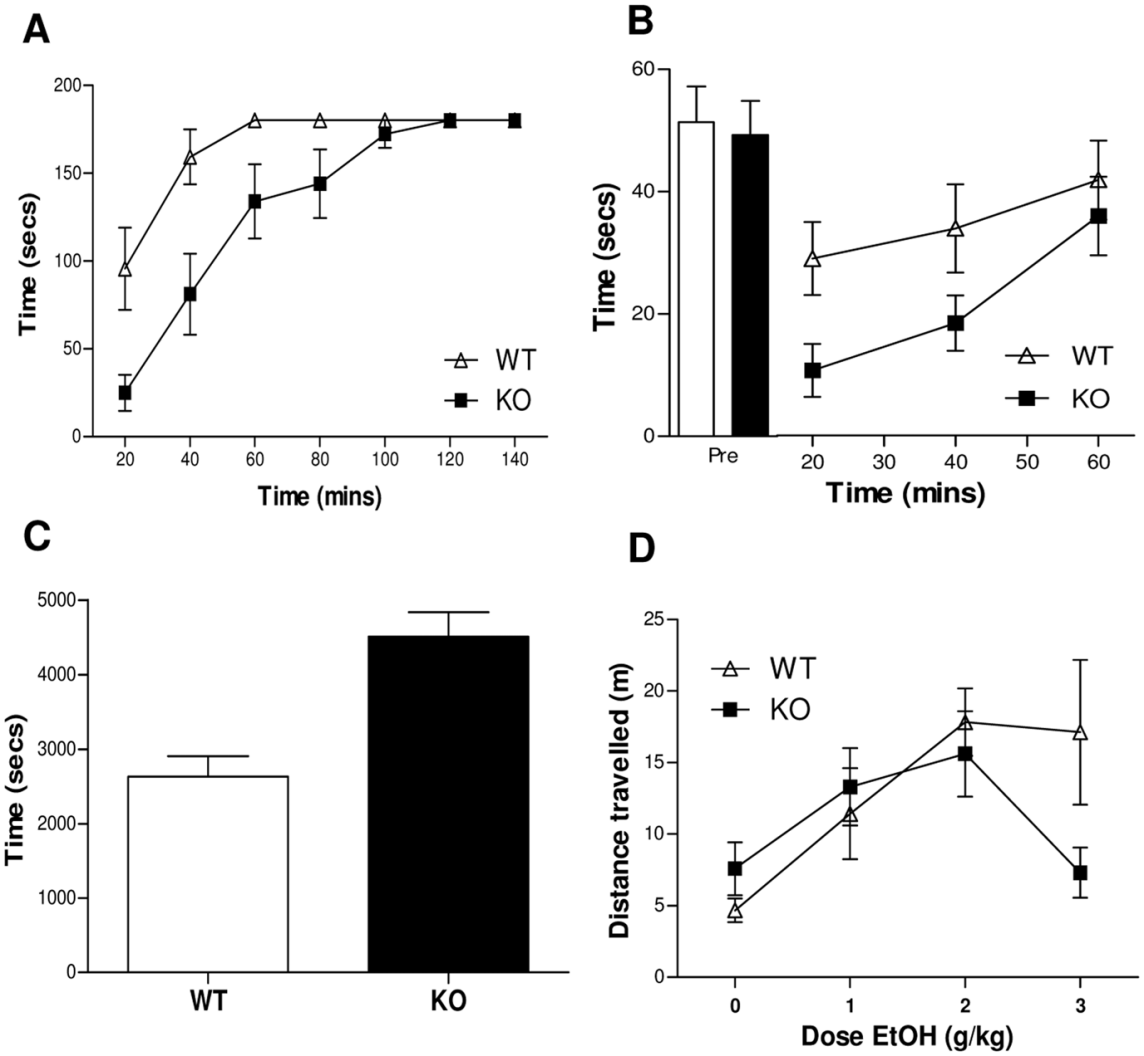
Increased ataxia and sedation after acute ethanol administration in GABA_A_ α2-subunit KO mice. (**A**) Latency (secs) to fall from the Rotarod apparatus. WT mice are able to remain on the Rotarod apparatus for longer than KO after 2 g/kg ethanol (n = 10; genotype by time interaction, F_(6,108)_ = 3.703, p<0.05, ε = 0.604) (**B**) Latency (secs) to fall from the wire hang apparatus. WT mice are able to retain a grip on the hanging wire longer than KO mice after 2 g/kg ethanol. (n = 12; genotype by time interaction, F_(2,44)_ = 3.133, p = 0.05) (**C**) Loss of righting reflex. KO animals show a longer duration of sedation after 3.5 g/kg ethanol (n = 12; t(22) = −4.4, p<0.001) (**D**) Locomotor dose response. KO animals show reduced locomotion at 5 mins after 3 g/kg ethanol when compared to WT animals (n = 8; genotype by dose interaction, F_(3,39)_ = 3.075, p<0.05).

Prior to receiving an ethanol injection, all animals were able to complete a 3 minute trial on the Rotarod apparatus. After administration of ethanol, both genotypes improved their performance over the course of the experiment (Main effect of time; F_(6,108)_ = 26.702, p<0.001, ε = 0.604; Fig. 1A). However, KO mice were more impaired in their performance (main effect of genotype, F_(1,18)_ = 13.775, p<0.01) and were slower to recover the ability to remain on the Rotarod for 180 seconds (genotype by time interaction, F_(6,108)_ = 3.703, p<0.05, ε = 0.604).

#### Wire hang

Prior to ethanol administration, both genotypes demonstrated a similar degree of motor strength and remained on the wire for a comparable amount of time. (t(22) = −0.007, p = 0.995; Fig. 1B). However, after 2 g/kg ethanol, KO mice showed a marked decrease in the time spent on the wire compared to WT (genotype by time interaction, F_(2,44)_ = 3.133, p = 0.05) but both genotypes recovered within a similar time frame.

#### Loss of righting reflex

After administration of 3.5 g/kg ethanol, KO mice showed increased sedation compared to WT (t(22) = −4.4, p<0.001), as measured by a longer duration of the loss of righting reflex (Fig. 1C).

#### Locomotor activity

No genotype differences were noted during habituation to the locomotor apparatus (genotype by dose interaction, F_(1,14)_ = 0.598, p = 0.452; main effect of genotype, F_(1,14)_ = 0.268, p = 0.613; data not shown). Locomotor activity at five minutes after ethanol administration was lower in KO mice compared to WT (genotype by dose interaction, F_(3,39)_ = 3.075, p<0.05; Fig 1D).

### Motivation to Obtain Ethanol

#### Operant self-administration

**Figure 2:**
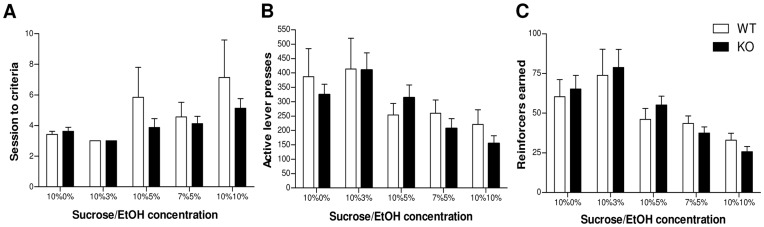
Self administration of sucrose/ethanol solution remains unchanged in GABA_A_ α2-subunit KO mice. (**A**) Sessions to criteria**.** The genotypes did not differ in their acquisition of self administration, performing a similar number of sessions to criteria at each concentration of ethanol (n = 8; 10%0%: U = 31.5, p = 0.648; 10%3%: U = 28.0, p = 1.00; 10%5%: U = 22.5, p = 0.448; 7%5%: U = 27.5, p = 0.951; 10%10%: U = 28.0, p = 1.00) (**B**) Number of active lever presses. Both genotypes perform a comparable number of lever presses to obtain increasing concentrations of ethanol in a sucrose/ethanol solution (genotype by reinforcer interaction, F_(4,52)_ = 0.721, p = 0.486, ε = 0.462) (**C**) Number of reinforcers earned. Both WT and KO mice earned a similar number of reinforcers at each concentration of ethanol (genotype by reinforcer interaction, F_(4,52)_ = 0.486, p = 0.604, ε = 0.456), indicating that motivation to obtain ethanol is unchanged after a deletion of the GABA_A_ α2-subunit.

The acquisition of self administration of sucrose and ethanol solutions did not differ between genotypes. Both genotypes performed a similar number of sessions to criterion at each concentration (10%0%: U = 31.5, p = 0.648; 10%3%: U = 28.0, p = 1.00; 10%5%: U = 22.5, p = 0.448; 7%5%: U = 27.5, p = 0.951; 10%10%: U = 28.0, p = 1.00; Fig. 2A). When allowed to lever press to gain access to increasing concentrations of ethanol in a sucrose solution, KO mice did not differ from WT in either the number of active lever presses (genotype by reinforcer interaction, F_(4,52)_ = 0.721, p = 0.486, ε = 0.462; main effect of genotype, F_(1,13)_ = 0.019, p = 0.893; Fig. 2B) or the number of reinforcers earned (genotype by reinforcer interaction, F_(4,52)_ = 0.486, p = 0.604, ε = 0.456; main effect of genotype, F_(1,13)_ = 0.034, p = 0.858; Fig. 2C) in 30 minutes. The genotypes performed to a similar level at all concentrations, both reducing their number of reinforcers as the ethanol concentration increased (Main effect of concentration, F_(4,52)_ = 12.357, p<0.001, ε = 0.456).

### Blood Ethanol Concentration

**Figure 3:**
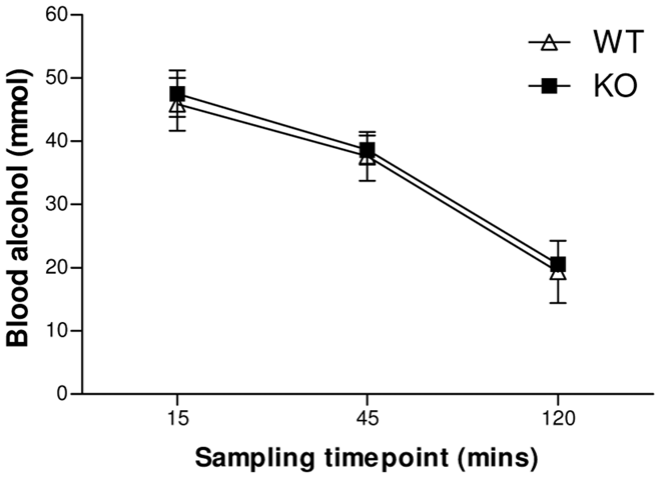
Blood ethanol concentration. Following an acute ethanol challenge (2 g/kg, i.p.), no genotype differences were observed in blood ethanol concentration (n = 4; genotype by sampling time interaction F_(2,15)_ = 0.005, p = 0.995).

A significant main effect of post-administration sampling time (F_(2,15)_ = 26.18, p<0.001; Fig. 3) indicates that amount of ethanol in the blood decreases over time. Both main effect of genotype (F_(1,15)_ = 0.191, p = 0.668) and genotype by sampling time interaction (F_(2,15)_ = 0.005, p = 0.995) were non-significant, indicating that the rate of ethanol metabolism is similar in both genotypes.

## Discussion

The current data suggest a role for the GABA_A_ α2-subunit in mediating the sedative and ataxic effects of acute ethanol administration. Deletion of this subunit resulted in a decreased ability to maintain walking on a Rotarod apparatus under ethanol treatment, decreased motor strength as measured in the wire hang test, and a greater duration of the loss of righting reflex. In response to an acute ethanol challenge, KO animals showed lower locomotor activity compared to WT at the highest dose, further suggesting increased sedation in the KO. It should be noted that locomotor activity was measured for five minutes immediately following ethanol injection, to capture the period during which ethanol is activating. Thus, the doses administered to induce locomotor activity may seem high in comparison to other publications that emphasise the sedative effects of the drug. Since metabolism was unaffected, the effects observed indicate an increased sensitivity to the sedative and ataxic effects of ethanol. In spite of the observed acute difference, no change in the motivation to consume an ethanol solution was observed, suggesting this increased sensitivity does not extend to the rewarding properties of ethanol.

GABA_A_ receptors are expressed throughout the CNS, with different receptor subtypes providing heterogeneous expression throughout distinct brain regions. Whilst the exact neural basis for the sedative and ataxic affects of ethanol is currently unknown, the α2-subunit is expressed within brain areas involved in motor output, namely the striatum, cerebellum [29], [30] and within the dorsal and ventral horns of the spinal cord [31], [32], making these the most likely loci to mediate acute sedative/ataxic effects.

There are several mechanisms by which a manipulation of GABAergic inhibition may affect responses to ethanol. Ethanol can activate postsynaptic receptors directly at high concentrations but whether this effect occurs at concentrations likely to be achieved socially is controversial [7]. Thus loss of GABA_A_ α2-subunits is unlikely to change ethanol responses in the current experiments which result in relatively low blood alcohol concentrations, but currently cannot be ruled out as a possibility. These lower concentrations, however, can induce release of neurosteroids, which are known to act at GABA_A_ receptors. The actions of neurosteroids have been shown to be only modestly influenced by the α-subunit composition of the receptor, with an alteration of the γ-subunit having the most effect [33]. Furthermore, changes in neurosteroid levels do not appear to alter the acute motor effects of ethanol [34], suggesting that a neurosteroid-dependent mechanism is unlikely to explain the acute differences observed in the current study. Increased GABA release also occurs in response to ethanol administration and is induced, at least in part, by a presynaptic GABA_A_ receptor mechanism [35], [36]. The α2-subunit has been found presynaptically in hippocampal mossy fibre neurons [37] and thus may serve to modulate GABA release. Since presynaptic GABA receptors attenuate ethanol-induced GABA release [36], it might be speculated that a deletion of the α2-subunit would result in increased GABA release in response to an ethanol challenge, causing an enhanced acute response. However, it is important to note that evidence of presynaptically expressed α2-subunits in brain regions other than hippocampus, and a direct link with ethanol effects remains to be demonstrated.

In spite of acute differences to the sedative and ataxic effects, motivation to drink as measured by operant self-administration of an oral ethanol solution remained unchanged in KO mice. The increased sensitivity to the motor effects of ethanol in the KO did not appear to affect the ability to obtain the solution since both genotypes self administered at a similar rate for all concentrations and neither genotype indicated an inability to perform due to inebriation. Importantly, the absence of a change in ethanol self-administration is consistent with previously published data using KO mice, which showed no differences in ethanol drinking preference as measured by a two-bottle choice paradigm [24]. However, a point mutation of the α2-subunit gave rise to varying effects on ethanol drinking depending on the precise test conditions, with the mutant drinking more alcohol during short-term daily access and less alcohol during continuous access [22]. Whilst these findings imply an involvement of the α2-subunit in ethanol drinking, they are difficult to interpret without further investigations of factors such as taste reactivity, withdrawal or motivational effects. Given the regional distribution of the α2-subunit, it is perhaps not surprising that motivated drinking, as measured by operant self-administration, is unaffected by its deletion. In contrast to psychostimulants, lesions of the striatum (where the α2-subunit is heavily expressed) do not affect motivation to consume ethanol [38], [39]. Instead, it has been suggested that the ventral pallidum may be more important in maintaining ethanol drinking, consistent with a role of the GABA_A_ α1-subunit [40], [41] which is more abundant within this region.

The lack of an effect of a *gabra2* deletion on drinking behaviour seems at first sight to be in disagreement with the human literature suggesting that polymorphisms of the α2-subunit are involved in ethanol dependence [1], [2]. Both risk and protective haplotypes have been identified, but it is currently unknown if and how these haplotypes translate into expression differences and if they would be in any way analogous to the constitutive mouse knockout. One factor that should be considered is how variations in the α2-subunit may interact with other intrinsic or environmental influences. Recent evidence suggests that the α2-subunit plays a role in ethanol dependence only in those who have been subjected to childhood trauma [11]. Since our mice received no developmental stress, it is perhaps unlikely that they would reveal a role of α2-subunit containing receptors in motivation to drink ethanol.

An important consideration of any experiment using a constitutive knockout model is that compensatory changes may take place in response to the deletion, potentially causing changes in receptor function that will have occurred throughout the brain and during development and may be responsible for the resulting changes in phenotype. Certainly, in the case of deletion of the *gabra1* gene resulting in loss of the α1-subunit, there is evidence for increased expression of α2- and α3-subunits, and decreased expression of γ2-subunits, as well as alterations in receptor clustering and distribution [42], [43] and in various other genes involved in neural plasticity [44]. Using quantitative PCR analysis, we found no evidence for altered expression of other GABA_A_ receptor subunits in the KO [10], indicating a lack of compensation in subunit expression at the transcription level in the adult mouse. Nevertheless we cannot exclude changes either at the protein level, or patterns of insertion of receptors into the membrane, or to other neurotransmitter systems.

Such compensatory changes may be important when comparing data from other mutant mouse models. Differing mutations of the α2-subunit have resulted in both increased sedation in response to an ethanol challenge (S270H, L277A mutant [22]) and decreased sedation (H101R mutant [45]). The difference between these studies and the current data may be explained by a difficulty in identifying the mechanism by which ethanol is affecting GABA_A_ transmission. Whether it is a direct mechanism or secondary effects via other neuromodulators, such as neurosteroids, manipulating different amino acid residues within the subunit may produce a variation in the receptor responses. However, it is noteworthy that a point mutation in α2-subunits (serine 270 to histidine and leucine 277 to alanine mutations) resulting in reduction of sensitivity to ethanol’s ability to facilitate GABA-induced current, when expressed in mice also enhanced ethanol’s effects on the loss of the righting reflex, without influencing ethanol consumption [22]. Since point mutations may be less likely to give rise to compensatory changes (but see [17]) than deletions, such a result would be consistent with the behavioural effects we observed being due to loss of α2-subunit function, and not to compensatory effects.

It is important to note that previous experiments using *gabra2* KO mice have shown a decrease in sensitivity to the acute effects of ethanol as shown by a reduced loss of righting reflex [24], the direct opposite response to the one reported here. The discrepancy between this experiment and the current data are not attributable to differences in generation of the knockout, as in both cases the mice were derived from the same original line [19], [24]. Furthermore, the two knockout colonies have both been maintained on the same background strain (50% C57BL/6J, 50% 126SvEv) which minimises the possibility of differences in epistatic interactions with genes from the background strain influencing the behaviour. At present, we can only suggest that differences may reflect consequences of genetic drift within two independently-maintained colonies.

In conclusion, the current data demonstrate that deletion of the *gabra2* gene encoding the GABA_A_ α2-subunit results in an increased sensitivity to the acute sedative and ataxic effects of ethanol administration, without changing the motivation to consume ethanol. In conclusion, these observations imply a protective role of the α2-subunit against the acute motor effects of high dose ethanol but the exact mechanism remains unclear. Whilst genetic variations of the GABRA2 gene in humans have previously implicated this subunit in ethanol abuse, current data suggest that this is unlikely to be due to GABAergic manipulations directly changing motivational behaviour. Considering the complexity of the literature regarding the potential role of the GABA_A_ α2-subunit in responses to ethanol, it may be important to consider the participation of intermediate messenger systems. In the case of a role for variations in α2-subunit containing receptors as risk factors for addiction, additional factors, such as stress, and how they interact with GABAergic inhibition may be important in producing addiction-related phenotypes.
